# Salinity in Drinking Water and the Risk of (Pre)Eclampsia and Gestational Hypertension in Coastal Bangladesh: A Case-Control Study

**DOI:** 10.1371/journal.pone.0108715

**Published:** 2014-09-30

**Authors:** Aneire Ehmar Khan, Pauline Franka Denise Scheelbeek, Asma Begum Shilpi, Queenie Chan, Sontosh Kumar Mojumder, Atiq Rahman, Andy Haines, Paolo Vineis

**Affiliations:** 1 MRC-HPA Centre for Environment and Health and Dept. of Epidemiology and Biostatistics, School of Public Health, Imperial College London, London, United Kingdom; 2 Grantham Institute for Climate Change, Imperial College London, London, United Kingdom; 3 Dhaka Children’s Hospital, Dhaka, Bangladesh; 4 Upazilla Health Complex Dacope, Khulna, Bangladesh; 5 Bangladesh Center for Advanced Studies, Dhaka, Bangladesh; 6 London School of Hygiene and Tropical Medicine, London, United Kingdom; Gentofte University Hospital, Denmark

## Abstract

**Background:**

Hypertensive disorders in pregnancy are among the leading causes of maternal and perinatal death in low-income countries, but the aetiology remains unclear. We investigated the relationship between salinity in drinking water and the risk of (pre)eclampsia and gestational hypertension in a coastal community.

**Methods:**

A population-based case-control study was conducted in Dacope, Bangladesh among 202 pregnant women with (pre)eclampsia or gestational hypertension, enrolled from the community served by the Upazilla Health Complex, Dacope and 1,006 matched controls from the same area. Epidemiological and clinical data were obtained from all participants. Urinary sodium and sodium levels in drinking water were measured. Logistic regression was used to calculate odds ratios, and 95% confidence intervals.

**Findings:**

Drinking water sources had exceptionally high sodium levels (mean 516.6 mg/L, S.D 524.2). Women consuming tube-well (groundwater) were at a higher disease risk than rainwater users (p<0.001). Adjusted risks for (pre)eclampsia and gestational hypertension considered together increased in a dose-response manner for increasing sodium concentrations (300.01–600 mg/L, 600.1–900 mg/L, >900.01 mg/L, compared to <300 mg/L) in drinking water (ORs 3.30 [95% CI 2.00–5.51], 4.40 [2.70–7.25] and 5.48 [3.30–9.11] (p-trend<0.001). Significant associations were seen for both (pre)eclampsia and gestational hypertension separately.

**Interpretation:**

Salinity in drinking water is associated with increased risk of (pre)eclampsia and gestational hypertension in this population. Given that coastal populations in countries such as Bangladesh are confronted with high salinity exposure, which is predicted to further increase as a result of sea level rise and other environmental influences, it is imperative to develop and evaluate affordable approaches to providing water with low salt content.

## Introduction

Hypertensive disorders in pregnancy are among the leading causes of maternal and perinatal death in low-income countries [1]. Of these, (pre)eclampsia is one of the top five causes of direct maternal deaths [1], [2], and is associated with perinatal death through increased risk of preterm birth and intrauterine growth restriction, together with higher childhood blood pressure (BP) in offspring, and future cardiovascular disease in mothers [3], [4].

There is a wealth of epidemiological evidence associating high salt intake with the risk of hypertension in children and adults [5]. However, the role of salt in the aetiology of hypertensive disorders in pregnancy remains largely unclear, according to a Cochrane review [6].

In a survey conducted in 2008, rates of (pre)eclampsia and gestational hypertension were found to be higher in Bangladesh’s coast compared to non-coastal areas [7]. The rates were also considerably high in the dry season, when salinity levels in surface and groundwater are higher than in the monsoon season [8], [9].

Bangladesh’s coastal population, comprising approximately 40 million people, relies heavily on natural water sources like ponds, rivers and tube-wells for obtaining drinking water. These sources have become severely saline from seawater intrusion caused by environmental changes, and man-made factors including poor water management and shrimp farming [9]. Salinity has already encroached >100 km inland from the Bay of Bengal, and the impacts are projected to be exacerbated by sea level rise due to climate change and excessive groundwater withdrawals from aquifers [10], [11].

In light of this, this epidemiological study was conducted to investigate the association between consumption of highly saline drinking water and the risk of (pre)eclampsia and gestational hypertension among pregnant women in coastal Bangladesh.

## Methods

### Study design and participants

A population-based case-control study was conducted between October 2009 and April 2011 in Dacope Upazilla, a rural coastal sub-district in the Khulna district of south-west Bangladesh, among a population of approximately 143,000 people.

Cases of (pre)eclampsia and gestational hypertension were obtained from the Upazilla Health Complex, Dacope (UHCD). Cases were also identified and recruited from the local community within the study area. Health assistants measured the BP of all identified pregnant women (aged 13–45) within Dacope, at gestation week 20 and onwards. In the presence of high BP (>140/90 mmHg) and/or oedema, they were referred to the UHCD, where diagnoses were made by study physicians. BP was measured in millimeters of mercury (mmHg) directly by manual sphygmomanometers, using a standard protocol [12].

Controls included pregnant women at gestation week 20 and onwards, without hypertension, randomly selected from the same study population, and within the same time frame as the selection of the cases. Four controls per case were selected.

The study protocol was approved by the ethics committee of the Bangladesh Medical Research Council (BMRC). All participants gave written informed consent before initiation of study activities.

#### Drinking water and confounders

Information on drinking water sources, previous pregnancies, socioeconomic conditions, occupation, lifestyle, diet, and medical histories was collected through interviews. The questionnaire was a modified version of the one used in the Health Effects of Arsenic Longitudinal Study (HEALS) cohort [13]. Study participants visited the UHCD, where study physicians completed the questionnaire’s clinical sections, measured BP again and proteinuria, and made disease diagnoses, if relevant. The study physicians were blind to the water sources. Definitions of the health outcomes are summarised in **Table 1**.

**Table 1:** Definition of health outcomes included in the study [33].

**Pre-eclampsia:**Raised BP of >140/90 mmHg and significant proteinuria (0.3 grams on at least two random clean catch urine samples), with or without pathological oedema, occurring after the 20th week of pregnancy, in a previously normotensive and non-proteinuric patient.
**Eclampsia:**Pre-eclampsia complicated with convulsions that could not be attributed to other causes and/or coma.
**Gestational hypertension:**Raised BP (>140/90 mm Hg) detected for the first time after mid-pregnancy and without proteinuria or pathological oedema.

#### Sodium measurements

Drinking water samples (250 ml) were collected during the time of the interview, and sent to Dhaka University for analysis. Sodium was measured by the Atomic Absorption method, using A Analyst 800, Perkin Elmer, USA, and expressed in milligrams per litre (mg/L).

To further check sodium intake and excretion, we collected urine samples amongst the controls who were given appropriate containers and instructions for collecting 24-hr urine samples. A laboratory technician homogenised the specimens, recorded the total volume, and stored 10 ml of each sample at 4 C.

Spot urine, instead of 24-hr urine, was collected from cases to reduce distress caused to the ill pregnant women. This was considered to be less intrusive for women who were unwell. However, because of lack of comparability with controls, these samples were not used in the case-control comparisons. Only controls samples were used to validate water measurements.

Urine samples were sent to the International Centre for Diarrhoeal Disease Research in Bangladesh (ICDDR, B) for analysis. Urinary sodium was measured by Ion Selective Electrode method (ISE), using Automated Chemistry Analyzer, Olympus, Model AU640, Beckman Culter International, Japan. Spot urinary excretion values were measured in millimoles per litre (mmol/L). Individual 24-hr sodium excretion values were calculated as the product of concentrations in urine and the total urine volumes, measured in millimoles per day (mmol/d).

### Statistical analyses

All statistical analyses were performed using Stata 11.0 for Windows. Water sources were grouped as: rainwater (salt-free), filtered pond water, pond water (unfiltered) and tube-well water. The reference category contained women who reported rainwater combined with those who reported ‘rainwater with another source’ due to low numbers in the ‘rainwater only’ group. Water sodium levels were divided into groups: <300.01 mg/L, 300.01–600 mg/L, 600.01–900 mg/L and >900.01 mg/L, based on the distribution among cases.

Analyses on (pre)eclampsia and gestational hypertension were carried out separately, as well as a single (combined) health outcome as they appear to have similar relationships with salt intake from drinking water.

Summary descriptive statistics for all continuous and categorical variables were calculated separately for cases and controls. Logistic regression was used to calculate the odds ratios (ORs), and corresponding 95% confidence intervals (CI) for the health outcomes in relation to water sources and sodium levels in drinking water. Multivariate regression analyses were adjusted by age, parity, and mid-upper arm circumference (as a proxy measure of adiposity; BMI was not used because of oedema in pre-eclamptic women) based on a priori knowledge about potential confounders. In the matched analysis, cases and controls were matched by age, parity and the area of residence within Dacope.

## Results

### Summary descriptive statistics

**Figure 1:**
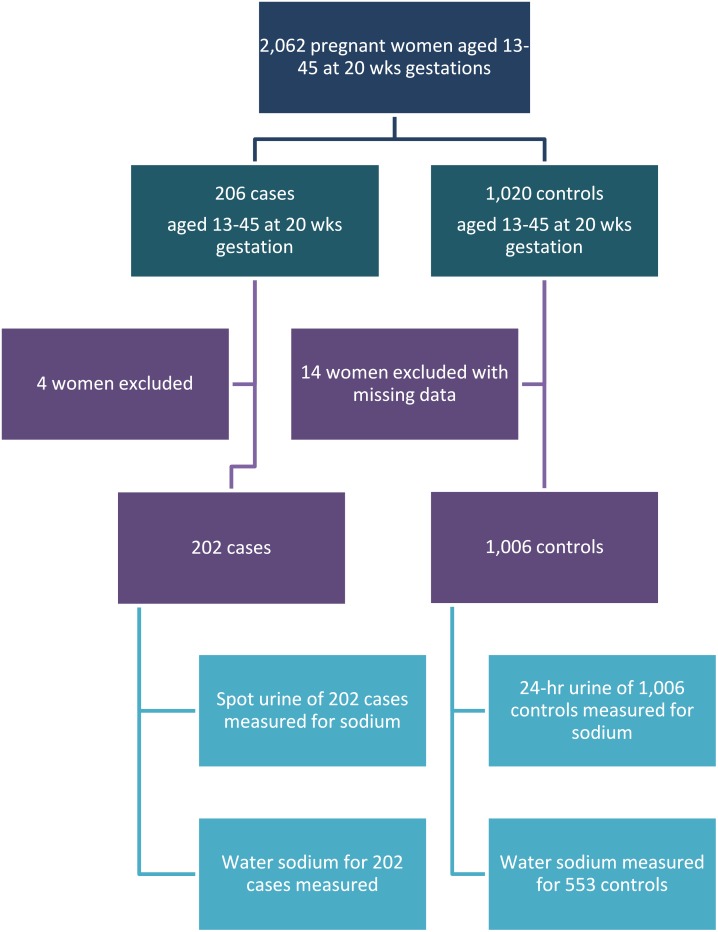
Recruitment of cases and controls.

We recruited 206 cases and 1,020 randomly selected controls. Four cases were excluded because they were known to have pre-existing hypertension. Among controls, women with missing data for urinary sodium (n = 2) and BP (n = 12) were excluded. In the final analysis, 202 cases and 1,006 controls were included. For the first nine months, drinking water sodium was analysed on all controls. For the subsequent nine months of the study, because of resource constraints, water sodium was analysed on a random sub-sample of the enrolled controls. Therefore, water sodium levels were measured for 202 cases and 553 controls (**Figure 1**).

There were 162 cases of pre-eclampsia, 33 cases of gestational hypertension, 7 cases of eclampsia. Since the sample population was measured for BP at the 20^th^ week of gestation, it was possible to diagnose and treat pre-eclampsia early, and disease progression into eclampsia reduced drastically, compared to previous years (personal communication from the UHCD).

The socio-demographic characteristics of the 202 cases and 1,006 controls, and the crude risk of the disease with different exposures are described in **Table 2**. Mean water sodium levels of drinking water sources were significantly higher in the cases (728 mg/L) than the controls (440 mg/L) (p<0.001) (**Table 3**). This difference was not significantly affected when the comparison was made separately for the first and second nine-month periods of the study.

**Table 2:** Summary descriptive statistics, mean (sd) or percentage (%) of cases and controls and crude risks with (pre)eclampsia and gestational hypertension.

Baseline characteristics	Cases (n = 202)	Controls (n = 1,006)	Odds ratio and 95% CI
**Age in years (mean, s.d.)**	23.8 (4.81)	23.1 (4.50)	1.03 (1.00–1.07)
**Age in categories, n, %**			
< = 19	40 (19.8)	245 (24.4)	1
19–24	79 (39.1)	424 (42.2)	1.14 (0.76–1.70)
25–29	53 (26.2)	240 (23.9)	1.35 (0.86–2.12)
>30	30 (14.9)	97 (9.64)	1.89 (1.12–3.33)
**Religion**			
Muslim, n, %	89 (44.1)	376 (37.4)	1
Hindu, n, %	111 (55.0)	603 (60.0)	0.78 (0.57–1.06)
Christian, n, %	2 (0.99)	25 (2.49)	0.34 (0.08–1.45)
Others, n, %	0 (0.00)	2 (0.20)	-
**Years in education, n, %**			
No education (0 y)	9 (4.50)	74 (7.40)	1
Up to primary school (6 y)	53 (26.2)	290 (28.8)	1.50 (0.71–3.19)
Incomplete secondary (10 y)	94 (46.5)	481 (47.8)	1.61 (0.77–3.32)
Secondary School (12 y)	31 (15.4)	86 (8.60)	2.96 (1.32–6.63)
Higher Secondary or above (>14 y)	15 (7.43)	75 (7.46)	1.64 (0.68–4.00)
**Socioeconomic status Index**			
Low	32 (15.8)	371 (36.9)	1
Middle	83 (41.1)	356 (35.4)	2.70 (1.75–4.17)
High	87 (43.1)	279 (27.7)	3.62 (2.34–5.58)
**Profession**			
Housewife	192 (95.1)	980 (97.4)	1
Service, business, daily labourer	10 (4.95)	26 (2.58)	1.96 (0.93–4.14)
**Previous children, n, %**			
Multiparous, n, %	81 (40.1)	503 (50.0)	1
Nulliparous, n, %	121 (60.0)	503 (50.0)	1.50 (1.10–2.03)
**Parity**			
No child, n, %	121 (60.0)	503 (50.0)	1
1 child, n, %	58 (28.7)	335 (33.3)	0.72 (0.51–1.01)
2 children, n, %	16 (7.92)	119 (11.8)	0.56 (0.32–0.98)
3 or more children, n, %	7 (3.47)	49 (4.87)	0.59 (0.26–1.34)

**Table 3:** Comparison of blood pressure, anthropometric variables and mean urine and drinking water sodium levels in cases and controls.

Baseline characteristics	Cases (n = 202)	Controls (n = 1,006)	p-value
**Systolic BP (mean, s.d.)**	158.5 (15.0)	103.3 (10.3)	<0.001
**Diastolic BP (mean, s.d.)**	99.8 (9.6)	66.9 (8.9)	<0.001
**Weight in kg (mean, s.d.)**	56.8 (8.80)	49.7 (7.20)	<0.001
**Height in m (mean, s.d.)**	1.52 (0.05)	1.53 (0.06)	<0.020
**Mid upper arm circum in cm (mean, s.d.)**	24.5 (2.60)	23.3 (2.30)	<0.001
**BMI (kg/m^2^)**	24.6 (3.67)	21.3 (2.80)	<0.001
**Urinary sodium mmol/L (mean, s.d.)** *	113.0 (60.6)	104.4 (117.0)	<0.001
**Water sodium mg/L (mean, s.d.)** **	n = 202	n = 553	<0.001
	727.9 (469.3)	439.4 (522.3)	

*Not directly comparable, but correlated. See text for further explanation.

**See text for explanation.

### Urinary sodium and blood pressure in controls

**Figure 2:**
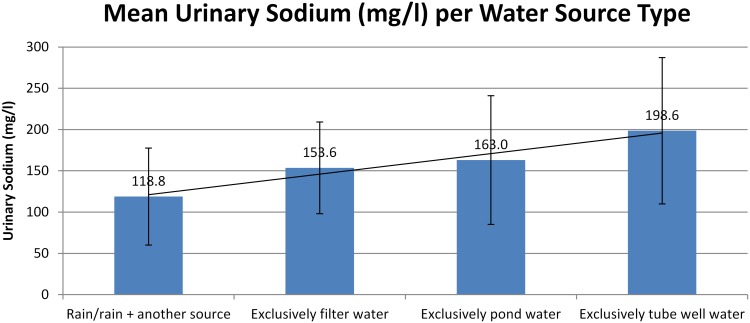
Mean 24-hr urinary sodium (mmol/d) in controls (n = 912) by water source.

In controls, drinking tube-well water was associated with the highest mean 24-hr urinary sodium excretion, compared to the other sources, while rainwater combined with another source had the lowest level (199 mmol/d for tube-well versus 119 mmol/d for rainwater; p<0.001) (**Figure 2**). A crude linear regression showed that women who drank filtered pond water, pond water or tube-well water had higher urinary sodium levels of 30.1 mmol/d (95% CI: 14.7–45.5), 37.5 mmol/d (95% CI: 25.0–50.0), and 60.0 mmol/d (95% CI: 48.1–71.7) respectively, than those who drank rainwater plus one other source (p<0.001).

Both systolic and diastolic BP in controls were associated with 24-hr urinary sodium excretion, showing a regression coefficient of 0.02 mmHg/mmol of sodium for systolic BP (p<0.001) and 0.01 mmHg/mmol of sodium for diastolic BP (p<0.001).

### Sodium levels by drinking water source

Tube-well water had the highest water sodium levels (714 mg/L), followed by filtered pond and pond water (**Table 4**).

**Table 4:** Mean water sodium levels (mg/L) in the drinking water of cases and controls combined among a sub-sample of women (n = 755) and estimated sodium intake (g/day).

Water source	n = 755	Mean water sodium (SD)	g/day with 2 L intake
**Rain only**	1	66.0	0.1
**Filter** 1 **only**	95	410.8 (410.0)	0.8
**Pond only**	231	374.3 (413.5)	0.7
**Tube-well only**	286	713.9 (533.0)	1.7
**River only**	8	136.2 (68.6)	0.3
**Multiple sources**	134	441.6 (625.5)	0.9
**All sources**	-	516.6 (524.2)	1.1

1 For brevity we refer to filtered pond water as ‘filter’.

### Blood pressure in all study participants by drinking water source

Both systolic BP and diastolic BP levels were associated with the type of drinking water source, with higher levels in sources that had higher salinity levels (p<0.001) (**Table 5**).

**Table 5:** Mean Systolic and Diastolic BP (mmHg) among cases and controls combined, by categories of water source.

Water source	n = 1,208	Mean Systolic BP	SD	Median
**Rain/rain+another**	244	102.4	16.2	100.0
**Filter only**	142	112.7	22.7	106.7
**Pond only**	298	112.6	21.8	108.3
**Tube-well only**	418	119.4	26.7	110.0
**Other**	106	108.7	20.4	100.0
***p-value<0.001***				
**Water source**	**n = 1,208**	**Mean Diastolic BP**	**SD**	**Median**
**Rain/rain+another** 1	244	66.2	12.0	66.7
**Filter only** 2	142	73.2	14.8	70.0
**Pond only**	298	72.6	13.9	70.0
**Tube-well only**	418	76.1	17.3	70.0
**Other** 3	106	70.5	12.9	70.0
***p-value<0.001***				

1 ‘Rain’ has been combined with any other water source because of small numbers in the rainwater only group.

2 For brevity we refer to filtered pond water as ‘filter’.

3 Those with multiple sources (except those who reported rainwater) and river have been grouped as ‘other’.

### (Pre)eclampsia and gestational hypertension risk and drinking water source

Among the 1,208 pregnant women, none of the cases reported rainwater as their only drinking water source, compared to 5 women in the control group (<1%) (**Table S1**). River water was excluded from the regression model (n = 14) as there were no cases in this group, and multiple sources (except those with rainwater and another source) were excluded as they cannot be classified and interpreted meaningfully (n = 92). The final model included age, parity, mid-arm circumference, and socioeconomic status as potential confounders.

The adjusted ORs for (pre) eclampsia and gestational hypertension among those who drank filtered pond, pond and tube-well water were 5.32 (95% CI 2.41–11.7), 5.31 (2.60–10.9) and 8.30 (4.20–16.4), compared to those who drank rainwater combined with another source (p<0.001) (**Table 6**).

**Table 6:** Association of (pre)eclampsia and/or gestational hypertension with water source.

Water Source	Cases(n = 202)	Controls(n = 1,006)	Crude Odds ratio (OR)(95% CI)	OR Adjusted by age, parity, SES,mid-upper arm circumference(95% CI)	P-value
Rain+another^1^	10 (5.26)	234 (25.7)	1.00	1.00	-
Filter^2^	25 (13.2)	117 (12.8)	4.99 (2.32–10.8)	5.32 (2.41–11.7)	<0.001
Pond	47 (24.7)	251 (27.5)	4.38 (2.16–8.87)	5.31 (2.60–10.9)	<0.001
Tube-well	108 (56.8)	310 (34.0)	8.15 (4.17–15.9)	8.30 (4.20–16.4)	<0.001

1 ‘Rain’ has been combined with any other water source because of small numbers in the rainwater only group.

2 For brevity we refer to filtered pond water as ‘filter’.

A sensitivity analysis by matching the area of residence (to control for unknown confounders), which included 147 cases and 147 controls, showed a similar adjusted risk trend with OR: 3.72 (95% CI 1.30–10.6) for women using filtered pond water, OR: 4.14 (95% CI 1.61–10.7) for pond water and OR: 4.60 (95% CI 1.61–10.7) for tube-well water (p<0.001).

Compared to the rainy season, the ORs for the different sources of water were consistently higher for the dry season, but the differences were not statistically significant (data not shown).

### (Pre)eclampsia and gestational hypertension risk and water sodium levels

**Figure 3:**
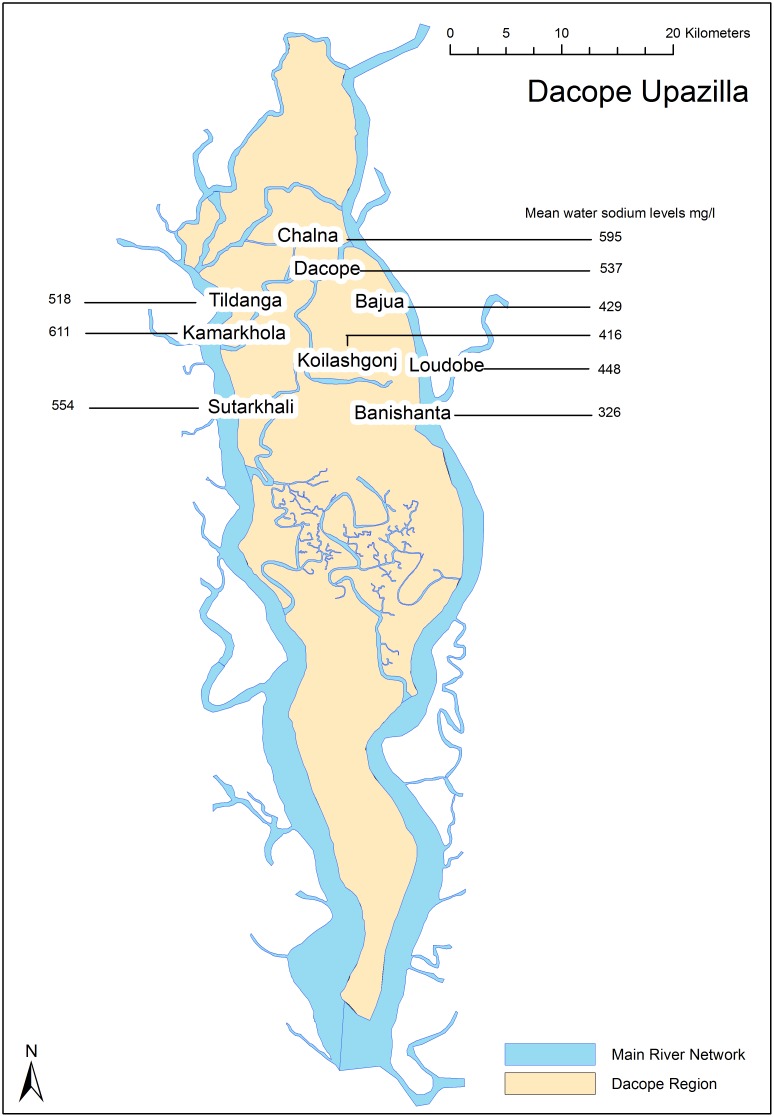
Map of Dacope sub-district showing the mean sodium levels of drinking water measured in various water sources.

Mean water sodium levels were universally high for all nine unions within Dacope (**Figure 3**). The adjusted ORs were 3.30 (95% CI 2.00–5.51), 4.40 (2.70–7.25) and 5.48 (3.30–9.11) for increasing sodium concentrations (OR = 1.0 for lowest; p<0.001) (**Table 7**). In the analysis including 147 cases and 147 controls with complete measurement of water sodium and matched by area of residence, the adjusted ORs were 2.40 (1.17–4.90), 6.23 (2.85–13.6) and 6.30 (2.90–13.6), p<0.001.

**Table 7:** Association of (pre)eclampsia and/or gestational hypertension with water sodium concentrations in drinking water.

Water sodiummg/L	Cases(n = 202)	Controls(n = 1006)	Crude Odds Ratio (OR)(95% CI)	OR Adjusted by age, parity, SES,mid-upper arm circumference (95% CI)	P-value
Min–300	43 (21.3)	277 (50.1)	1.00	1.00	-
300.01–600	45 (22.3)	106 (19.2)	2.73 (1.70–4.40)	3.30 (2.00–5.51)	<0.001
600.01–900	55 (27.2)	97 (17.5)	3.65 (2.30–5.80)	4.40 (2.70–7.25)	<0.001
900.01–max	59 (29.2)	73 (13.2)	5.21 (3.25–8.33)	5.48 (3.30–9.11)	<0.001

The adjusted ORs for pre-eclampsia cases only in relation to increasing sodium (300.01–600 mg/L, 600.1–900 mg/L, >900.01 mg/L, compared to <300 mg/L) in drinking water were 3.03 (95% CI 1.80–5.25), 4.12 (95% CI 2.40–7.00) and 4.60 (95% CI 2.66–7.90) (p<0.001). For gestational hypertension only, the adjusted ORs were 5.95 (95% CI 1.66–21.4), 7.00 (95% CI 2.00–24.3), and 13.4 (95% CI 4.17–43.0) (p<0.007).

## Discussion

Our study provides a novel insight into understanding the aetiology of hypertensive disorders in pregnancy among pregnant women living in coastal Bangladesh. This population is exposed to exceptionally high levels of sodium in drinking water, as shown by measurements in water and urine.

A positive association was found between salinity in drinking water and the risk of both (pre)eclampsia and gestational hypertension. A significant dose-response relationship was found with drinking water sodium levels, which persisted after adjusting for multiple potential confounders, and in the analysis matched by the area of residence. We observed a stronger association in the dry season but not statistically significantly so. These findings are consistent with a previous observation of an excess of (pre)eclampsia and gestational hypertension in the dry season, when salinity levels in surface and groundwater are higher than in the monsoon season [8], [9].

Preeclampsia and gestational hypertension risks were also independently associated with water sodium levels. Similarities in the patterns of risk factors indicate that at least some of the biological mechanisms underlying the two conditions may be similar.

Despite having the highest water sodium level, tube-wells were the commonest source of drinking water, implying that there is an urgent need for promoting alternative sources. The mean water sodium level found in all the water sources combined was 516 mg/L. Assuming an intake of 2 L, it is equivalent to 1.1 g/day of sodium intake from drinking water alone, which is >27 times higher than the intake from the recommended sodium limit of 20 mg/L in drinking water (equivalent to 0.04 g/day intake) set by the US Environmental Protection Agency [14]. The mean water sodium levels from drinking water alone, detected in this population, contribute to almost 52% of the dietary goal of 2 g/day set by the WHO [15]. Moreover, 38% of pregnant women consumed >1.2 g/day of sodium from drinking water, showing that a large number of people in this population are exposed to unacceptable levels of sodium.

The INTERSALT study reported that a median sodium excretion of >100 mmol/d over 30-years was associated with an increase of systolic BP by 3.1–6.0 mm Hg and diastolic BP by 0.1–2.5 mmHg, after adjusting for BMI [16]. In the present study, the mean 24-hr urinary sodium among the healthy pregnant women was 164 mmol/d and the median was 155 mmol/d, both of which are well above the recommended daily sodium intake of <85 mmol/d (or 2 g/day of sodium) [15]. Using the conservative BMI-adjusted estimates of the INTERSALT study, the present study’s mean intake of 164 mmol/d would increase systolic BP by 6.0–12 mmHg and diastolic BP by 0.2–5 mmHg, potentially leading to a large num­ber of pregnant women becoming hypertensive.

Direct comparison of urinary sodium between cases and controls is complicated by the use of spot urine for the cases and 24-hr urine for the controls, although these are known to be closely correlated [17]. The urinary sodium-BP relationship in the controls in the present study (coefficient = 0.02 mmHg/mmol) relates well with that found in the INTERSALT study where the systolic BP-sodium coefficient was 0.035 mmHg/mmol sodium and 0.015 for diastolic BP in non-pregnant adults [18], which strengthens the validity of the findings.

### Limitations of the study

We cannot exclude the possibility that some of the cases in the study had pre-existing hypertension. However, we excluded pregnant women (n = 4) who were known to have essential hypertension, which is uncommon among young women in rural Bangladesh. We also repeated the analysis excluding pregnant women >30 years of age in whom pre-existing hypertension may be more common; however, the results remained the same.

Urinary sodium between cases and controls could not be directly compared because of the different approaches used in collecting the samples (24-hr urine versus spot urine). Furthermore, sodium handling in cases and controls was likely to be different because of disease. The literature suggests that the capacity to excrete sodium is compromised in women with pre-eclampsia and gestational hypertension compared with normal pregnancy, and therefore excretion values do not always indicate correctly the amount of sodium intake [19]. Urine samples were collected mainly to demonstrate the association with sodium measurements in water, and thus the controls were used to fulfill this aim.

Water sodium samples could not be collected for all controls in the last nine months of the study, but rather from a random sub-sample of the enrolled controls (n = 553). This was mainly due to resource constraints. However, we conducted a matched analysis to ensure that this did not affect the results, and also found that the differences in cases and controls did not vary over the period of the study; therefore, the results are considered to be robust.

### Previous evidence and pathophysiology

It is well established that dietary sodium is a major risk factor for hypertension in children and adults and contributes significantly to the risk of death and disability from cardiovascular diseases, accounting for approximately 62% of strokes and 49% of coronary heart disease in high-income countries [5]. The sodium-BP relationship in pregnancy, however, remains unclear, with studies showing conflicting results [6]. The role of excessive salt intake in the aetiology of (pre)eclampsia and gestational hypertension is even less clear.

Two prospective studies conducted in Massachusetts [20] and Chicago [21] indicated that both systolic and diastolic BP were higher in groups with higher sodium exposure compared to low sodium in drinking water. A cross-sectional study in Arizona showed no difference in the prevalence of BP between those who consumed high- versus low-sodium drinking water [22]. However, the highest water sodium levels studied in the latter was much lower than the mean level reported in the present study.

High sodium intake was associated with the onset of pre-eclampsia by Lauro et al. [23]. A multicentre case-control study in Colombia showed that a dietary sodium intake of >2200 mg/day increased pre-eclampsia risk (OR: 3.18, 95% 1.19–8.48) [24]. A case-control study in Cairo, Egypt showed that a salty diet was associated with a higher preeclampsia risk (OR: 1.99, 95% CI: 1.02–3.91) [25]. A Cochrane systematic review that included only two trials with 603 pregnant women concluded that the evidence was inadequate to provide unequivocal information about the effects of a low sodium diet [6]. It is important to note that the present study addresses a population exposed to environmental conditions causing them to consume sodium from drinking water at much higher levels (>25 times) than most Western populations where the trials have been conducted [14].

Oxidative stress is proposed to play a major role in inducing endothelial dysfunction and the consequent clinical manifestations of pre-eclampsia and hypertension, with studies showing low antioxidant capacity and increased oxidative stress in hypertensive individuals and pre-eclamptic mothers [26]. Meanwhile, salt has been shown to induce oxidative stress in salt-sensitive individuals [27]. Several mechanisms control the sodium balance in the body, such as the renin-angiotensin-aldosterone system, and a disruption of their physiological functions may lead to the development of salt sensitivity [28]–[30]. The nutritional status of women in Bangladeshi women in rural areas is sub-optimal, and it is possible that excessive sodium exposure triggers the onset of preeclampsia in women with low antioxidant status [31].

### Conclusions

The present case-control study strongly suggests that high levels of sodium may be intimately involved in the causal pathway of (pre)eclampsia and gestational hypertension. Given the considerable burden of these diseases in the coastal areas of Bangladesh, and the associated adverse maternal and fetal outcomes, it is imperative to develop and evaluate affordable approaches to providing water with low salt content such as rainwater harvesting. A 2°C rise in sea-surface temperature and 0.3 metre sea-level rise is predicted to increase flood risk area in Bangladesh by 15% more than the present risk area, and depth of flooding by 23% within 20 km from the coastline [32]. It is likely that climate change effects will considerably exacerbate the current situation, which is likely to affect many coastal populations in low-income settings, and this adds further impetus to the need for intervention.

## Supporting Information

Table S1 **Main sources of drinking water for all study participants.**
(DOCX)
