# Supplementary material for: Salinity in Drinking Water and the Risk of (Pre)Eclampsia and Gestational Hypertension in Coastal Bangladesh: A Case-Control Study
Source: PLoS One. 2014 Sep 30;9(9):e108715. doi: 10.1371/journal.pone.0108715 (PMC4182542; doi:10.1371/journal.pone.0108715)
Supplement: Table S1 — Main sources of drinking water for all study participants. (DOCX) [file pone.0108715.s001.docx]

**Table S1: Main sources of drinking water for all study participants**

| **Water source** | **Cases (n=202)** | **Controls (n=1,006)** | **Total (n=1,208)** |
| --- | --- | --- | --- |
|  | **n (%)** | **n (%)** | **n (%)** |
| Rain only | 0 (0.00) | 5 (0.50) | 5 (0.41) |
| Filter only | 25 (12.4) | 117 (11.6) | 142 (11.8) |
| Pond only | 47 (23.3) | 251 (24.9) | 298 (24.7) |
| Tube-well only | 108 (53.5) | 310 (30.8) | 418 (34.6) |
| River only | 0 (0.00) | 14 (1.39) | 14 (1.16) |
| Multiple sources | 22 (10.9) | 309 (30.7) | 331 (27.4) |

*P-value <0.001*

1. ‘Multiple sources’ refers to those who reported more than one source
2. For brevity we refer to filtered pond water as ‘filter’
